# Signs of Disease

**Published:** 1881-07

**Authors:** 


HALL'S

### JOURNAL OF HEALTH

AND

MISCELLANY.

E. H. GIBBS, A. M., M. D. - - EDITOR.

Founded in 1854, and published Monthly without Interruption since that Time.

### SIGNS OF DISEASE.

IT is not always wise to inform a nervous patient of his condition, if he is suffering from incurable disease; but if information in regard to the symptoms that characterize the approach of those diseases that frequently prove fatal from neglect were generally disseminated all prudent persons would endeavor to avoid them by attending to these warnings.

It is just as absurd to expect that your physician can protect you from accident and disease, as that a church membership secures your salvation. Every man must possess the knowledge necessary to take care of himself, or he will sooner or later pay the penalty of his ignorance. This dependence on others is an evil of great magnitude in many ways. Life is neither profitable nor enjoyable unless mind and body are sound; and the best safeguard against diseases and accidents is the knowledge requisite to meet them, or to detect false alarms.

I have frequently referred to imaginary cases of heart disease, and I could perhaps render no greater service to many of the readers of the JOURNAL OF HEALTH than by stating a few facts in relation to the disease, where it actually exists; and here the importance of early and accurate knowledge, to the patient, is of incalculable value; because knowing his condition he will be enabled so to live as to maintain his general health, and render it more than probable that when he dies it will be from some other cause. I was acquainted with a gentleman in New York who suffered from serious heart disease during thirty years, and who died at eighty-four, from other causes. About nine persons in ten who come to consult me, think they have heart trouble. I have found these opinions justified in perhaps one case in fifty. This will not be wondered at when it is considered that dyspepsia, nervous excitement, care, anxiety and over fatigue usually produce more or less palpitation of the heart; and that this functional derangement is quite generally supposed to indicate heart disease, whereas it is usually of little moment. I never liked the idea of making a mystery of anything so simple as determining the existence or non-existence of heart disease. Almost any intelligent person can settle that question in any particular case in five minutes

The heart is a force-pump whose office is to circulate the blood. It is provided with chambers and valves like pumps of human construction. If these valves get out of order so that they do not close over the orifices tightly, a portion of the blood is forced back when the heart contracts, and this causes an irregular or peculiar pulse. It also produces certain characteristic sounds, which it is not necessary to notice here. If this irregularity of the pulse is constant it is more than probable that organic disease of the heart exists. If in addition to this there is considerable swelling of the feet, of the sort that retains indentations made with the finger, and that has a "doughy" feeling, it is probable that the disease of the heart has advanced considerably. This latter condition is also a well marked symptom of "Bright's disease" of the kidneys. The next marked symptom of heart disease, is shortness of breath; but this is also a sympton of other diseases. The pulse, then, is the most reliable indication of the heart's condition. It matters not how it flutters at times; so long as it beats naturally and regularly during sixty seconds at a time it is hardly possible that there should be any serious heart trouble. When disease exists there is a change of structure which makes regular pulsation impossible.

While on this subject, I will refer to another disease of which the pulse is an infallible indication; and that is in consumption. When there is emaciation, with the pulse ranging regularly from 90 to 120 beats a minute, no further evidence is necessary to confirm the fact that there is no hope of recovery.

A little timely knowledge would save a world of misery to most of these sufferers, beside the care, anxiety and grief of friends. Were one per cent. of the money now wasted in the purchase of trashy books and papers, used in learning how to live wisely, there would be an incalculable increase in the general happiness and length of life of the people.

---

"Dear Augustus," said an affectionate girl, "I am willing to marry you if we have to live on bread and water." "Well," responded the enthusiastic Augustus, "you furnish the bread, and I'll skirmish round and find the water."

